# A Sensor Array Based on Molecularly Imprinted Polymers and Machine Learning for the Analysis of Fluoroquinolone Antibiotics

**DOI:** 10.1021/acssensors.2c01260

**Published:** 2022-10-25

**Authors:** Mingyue Wang, Xavier Cetó, Manel del Valle

**Affiliations:** Sensors and Biosensors Group, Department of Chemistry, Universitat Autònoma de Barcelona, Faculty of Sciences, 08193 Bellaterra, Barcelona, Spain

**Keywords:** molecularly imprinted polymers, electronic tongues, artificial neural networks, fluoroquinolones, antibiotics, pharmaceutical analysis

## Abstract

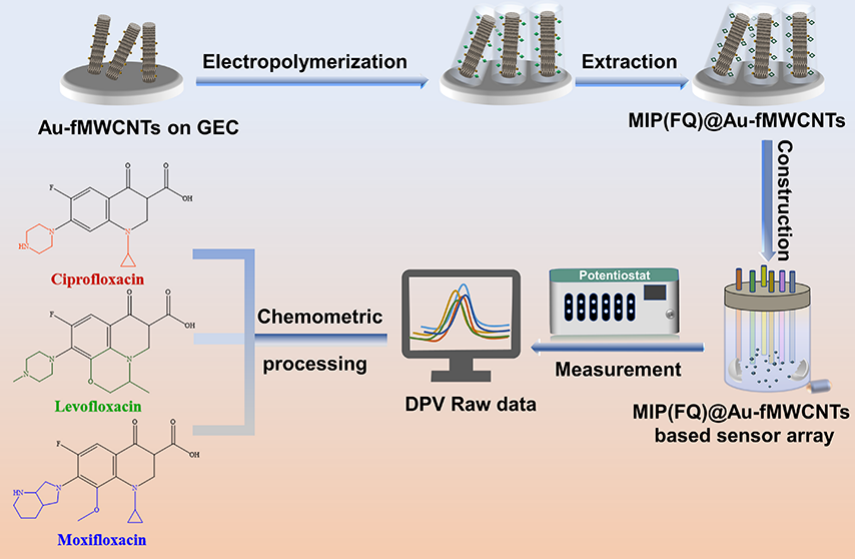

Fluoroquinolones (FQs) are one of the most important types of antibiotics in the clinical, poultry, and aquaculture industries, and their monitoring is required as the abuse has led to severe issues, such as antibiotic residues and antimicrobial resistance. In this study, we report a voltammetric electronic tongue (ET) for the simultaneous determination of ciprofloxacin, levofloxacin, and moxifloxacin in both pharmaceutical and biological samples. The ET comprises four sensors modified with three different customized molecularly imprinted polymers (MIPs) and a nonimprinted polymer integrated with Au nanoparticle-decorated multiwall carbon nanotubes (Au-fMWCNTs). MWCNTs were first functionalized to serve as a supporting substrate, while the anchored Au nanoparticles acted as a catalyst. Subsequently, MIP films were obtained by electropolymerization of pyrrole in the presence of the different target FQs. The sensors’ morphology was characterized by scanning electron microscopy and transmission electron microscopy, while the modification process was followed electrochemically step by step employing [Fe(CN)_6_]^3–/4–^ as the redox probe. Under the optimal conditions, the MIP(FQs)@Au-fMWCNT sensors exhibited different responses, limits of detection of *ca*. 1 μM, and a wide detection range up to 300 μM for the three FQs. Lastly, the developed ET presents satisfactory agreement between the expected and obtained values when used for the simultaneous determination of mixtures of the three FQs (*R*^2^ ≥0.960, testing subset), which was also applied to the analysis of FQs in commercial pharmaceuticals and spiked human urine samples.

Fluoroquinolones (FQs), one of the most important families of antibiotics, are used individually or in combination against a broad spectrum of Gram-positive and Gram-negative bacteria in clinical and animal husbandry.^1,2^ Among them, ciprofloxacin (CFX), levofloxacin (LFX), and moxifloxacin (MFX) are the most common in current clinical practice. LFX and MFX are considered as respiratory drugs that have good bactericidal activity against most of the respiratory pathogens, while CFX is used to treat the infections of the urinary tract and intestine. Structurally, FQs are similar as they are all derived from nalidixic acid (NA), sharing the same quinolone ring, but with different substitutions. Although the usage of FQs is approved worldwide and their significant curative effects have been demonstrated, consequences resulting from their misuse such as antibiotic residues (mainly related to food safety and environmental contamination) or antimicrobial resistance (essentially requiring therapeutic drug monitoring) cannot be neglected.^3−5^ Thus, FQs monitoring is required in different stages such as in pharmaceutical factories when produced, in hospitals during its administration to the patients, or in the environment for the treatment of wastewater.

To date, the detection of FQs using electrochemical sensors has been proposed as a cheaper and time- and labor-saving alternative to complex detection methods such as high-performance liquid chromatography or gas chromatography–mass spectrometry.^6^ Nevertheless, most reported electrochemical sensors target only one specific FQ, and cannot achieve the simultaneous multidetermination in practical applications or deal with the interferences that may arise from certain complicated matrixes.^7,8^ This can be rather thorny in the case of the FQ family given the common shared structure and similar natures.

Compared with the conventional single sensor approach, by coupling a sensor array providing responses to various analytes with appropriate data processing, electronic tongues (ETs) allow the discrimination and simultaneous quantification of multiple analytes,^9,10^ while they might also be able to solve other problems related to sensors such as drifts, interferences, cross-sensitivity, and/or matrix effects.

To further improve the performance of ETs, one of the trending strategies is using a proper set of biorecognition elements such as enzymes, peptides, etc.^11^ Recently, the feasibility of incorporating molecularly imprinted polymers (MIPs) into ETs has been investigated for the simultaneous determination of structurally similar analytes in various fields.^12,13^ As artificial customized receptors, MIPs not only can afford similar specificity to a bioreceptor but also can offer higher physical and chemical stabilities than biological recognition elements.^14,15^ More importantly, the host-guest recognition mechanism between MIPs and the target compounds, defined by the size and structure of the template molecules, offers a simple and straightforward procedure for developing a series of MIP-based sensors to be combined into a sensor array for the development of ETs. However, despite MIPs’ many merits, there is still room for improvement on their integration into sensors, especially in regard to sensitivity.^16^ Compared with other polymerization methods, electropolymerization can generate controllable MIP thin films directly onto transducer surfaces, representing a simpler and highly reproducible approach for the integration of MIPs with electrochemical sensors.^17,18^ In addition, the lower sensitivity derived from MIPs’ intrinsically inferior conductivity and electrocatalytic activity could be improved by introducing nanomaterials,^19^ for example, carbon nanotubes (CNTs)^20^ or metallic nanoparticles (NPs).^21^

To this end, herein we explore the electropolymerization of pyrrole (Py) onto multiwall CNTs (MWCNTs) as a supporting substrate that can lead to MIPs with a larger surface area, higher conductivity, and amplified signal. In addition, the incorporation of metallic gold NPs (Au NPs) can also act as a catalyst for the electrochemical reactions and further enhance the sensitivity of the obtained sensors. In this manner, MIPs integrated with these nanomaterials were synthesized toward CFX, LFX, and MFX. Next, the developed sensors were characterized both morphologically and electrochemically and finally combined into a sensor array, which was successfully employed to develop an MIP-based ET for the analysis of FQ antibiotics in commercial drugs and spiked human urine samples.

## Experimental Section

### Materials and Reagents

Graphite powder (particle size <50 μm, obtained from BDH, Poole, UK) and an epoxy resin kit (supplied by Resineco, Barcelona, Spain) were used for fabricating the graphite–epoxy composite (GEC) electrodes. Pristine MWCNTs (outer diameter ∼ 10 to 30 nm) were obtained from SES Research Inc. (Houston, TX, USA). Py and anhydrous trisodium citrate were purchased from Alfa Aesar (Ward Hill, MA, USA). Gold(III) chloride solution in hydrochloric acid (30 wt % HAuCl_4_ in diluted HCl), *N*,*N*-dimethylformamide (DMF), perfluorinated resin solution containing Nafion, CFX, LFX, MFX, flumequine (FLQ), and NA were purchased from Sigma-Aldrich (St. Louis, MO, USA), while sodium borohydride, potassium hexacyanoferrate(II) trihydrate, and potassium hexacyanoferrate(III) were purchased from Panreac (Barcelona, Spain). Ethanol (EtOH, 96 vol %) and NaOH were bought from Scharlau (Barcelona, Spain).

All solutions employed in the experiments were prepared using deionized water from a Milli-Q System (Millipore, Billerica, MA, USA). Various buffer solutions were used in this study for different purposes. Concretely, 50 mM phosphate buffer saline (PBS) at pH 7.0 containing 100 mM potassium chloride was used for the electrochemical characterization, while 100 mM PBS at pH 10.0 was used for the electrochemical extraction and cleaning. Then, 100 mM acetate buffer at pH 3.5 was used for the electropolymerization and preparation of the stock solutions, while 100 mM acetate buffer at pH 4.5 was employed for the analysis of FQs.

### Apparatus

An EVO MA10 (Zeiss, Oberkochen, Germany) and a JEOL 1210 (Peabody, MA, USA) were used for scanning electron microscopy and transmission electron microscopy (SEM/TEM) analyses, respectively. An Autolab PGSTAT30 potentiostat (Ecochemie, Utrecht, the Netherlands) with a multichannel configuration controlled by GPES and FRA software packages was used for the electrochemical measurements.

### Fabrication of the MIP(FQs)@Au-fMWCNT Sensor

Handcrafted GEC electrodes, which served as sensor platforms for the construction of the MIP-based sensor array, were prepared according to established procedures in our laboratory.^22^ Briefly, the paste was prepared by mixing 58% of graphite powder and 42% of epoxy resin. Next, this paste was placed into a PVC tube, in which a copper disc soldered to a connector had previously been inserted, and cured at 40 °C for 2 days. Finally, the electrodes were polished and ready to use.

Pristine MWCNTs were pretreated with strong acids to obtain carboxylic-functionalized MWCNTs (fMWCNTs). First of all, MWCNTs were dispersed in an acid mixture of concentrated H_2_SO_4_/HNO_3_ (3:1 v/v ) by ultrasonication for 6 h at 40 °C. Then, they were washed with Milli-Q water until neutralized and dried in an oven for further use.

The decoration of fMWCNTs with Au NPs was performed according to the previously reported procedure.^23^ Specifically, 500 μL of 10 mM HAuCl_4_, 500 μL of 10 mM trisodium citrate, and 18.4 mL of Milli-Q water were mixed in a round-bottom flask, to which 20 mg of fMWCNTs and 10 mL of anhydrous ethanol were added. After sonicating for 10 min, 600 μL of a 100 mM freshly prepared cold NaBH_4_ solution was also added into the mixture. The reaction lasted for 10 h under continuous stirring. The obtained product (denoted as Au-fMWCNTs) was thoroughly washed with Milli-Q water and dried in an oven at 40 °C overnight.

For the modification of the sensors, 2.0 mg of the dried Au-fMWCNT powder were dispersed in 1 mL of DMF into which 100 μL of Nafion were added to improve the stability of Au-fMWCNTs on the surface of the electrodes upon drop-casting. Finally, 4 μL of the Au-fMWCNT dispersion in DMF were evenly dropped onto the surface of the GEC and dried in the oven.

The synthesis of the MIP(FQs) film on the surface of Au-fMWCNTs was performed using the electropolymerization method. The solution containing 0.05 M Py as the functional monomer, 0.01 M FQ (either CFX, LFX, or MFX) as the template molecule, and 20 mL of acetate buffer (pH 3.5) as the supporting electrolyte was purged with nitrogen for 10 min before being used for the synthesis of the MIP(FQ) film. Electropolymerization was conducted by cycling the potential from 0.0 to +1.3 V versus Ag/AgCl at a scan rate of 50 mV·s^–1^(Figure S1). Subsequently, extraction of the template molecules from the polymer film was carried out in a two-step procedure: cycling the potential from 0.0 to +1.5 V versus Ag/AgCl at a scan rate of 100 mV·s^–1^ in PBS (pH 10.0) (Figure S1), followed by the immersion of the electrodes into a mixture of EtOH/0.1 M NaOH (1:1 v/v) for 30 min.

Similarly, the same procedures as abovementioned were used for the synthesis of the nonimprinted polymer on Au-fMWCNTs (NIP@Au-fMWCNTs), except that no FQ was added during electropolymerization. The purpose of the NIP is to be used as a control.

### Electrochemical Measurements

All electrochemical measurements were performed at room temperature employing a standard three-electrode cell made up of a Ag/AgCl (3 M KCl) reference electrode, a Pt wire as the auxiliary electrode, and the MIP(FQs)@Au-fMWCNT- or NIP@Au-fMWCNT-modified GECs as the working electrode. Both cyclic voltammetry (CV) and electrochemical impedance spectroscopy (EIS) for electrochemical characterization were conducted in a 5 mM [Fe(CN)_6_]^3−/4−^ solution in PBS (pH 7.0). For CV measurements, the potential was scanned in the range from −0.2 to +0.7 V at a scan rate of 50 mV·s^–1^, while for EIS, a frequency range from 100 kHz to 100 mHz with a fixed AC amplitude of 10 mV and an applied potential of +0.24 V was considered.

For the analysis of FQs, differential pulse voltammetry (DPV) measurements were performed by scanning the potential from +0.6 to +1.5 V with a step potential of 5 mV and a pulse amplitude of 50 mV without stirring. Prior to each measurement, the electrodes were incubated in the solution to be analyzed for 300 s under stirring to enhance the enrichment of the analytes into the MIP material. To regenerate the electrodes, a fixed potential of +1.2 V was applied for 90 s in PBS (pH 10.0) after each measurement.

### Sample Preparation

Given the different solubilities of the different FQs, the stock solutions of CFX, LFX, MFX, and NA were prepared in acetate buffer (pH 3.5), while the stock solution of FLQ was prepared in 0.1 M NaOH solution. In order to characterize the analytical response of the developed sensors toward each of the analytes, individual calibration curves were constructed by measuring solutions of increasing concentrations prepared by proper dilution of the FQs’ stock solutions in buffer (pH 4.5).

For the simultaneous quantification of CFX, LFX, and MFX, two subsets of samples were prepared and measured under identical conditions in a randomized order. On the one side, the training subset was composed by 27 samples based on a 3^3^ tilted factorial design^24^ in which the concentrations of FQs were varied in the range from 2 to 300 μM for CFX, LFX, and MFX. On the other side, to evaluate the performance of the proposed model, the testing subset was composed by 10 samples that were randomly distributed in the concentration domain defined by the factorial design.

To demonstrate its applicability, the MIP-based ET was applied to the determination of the FQs under study both in commercial pharmaceuticals and biological fluids. On the one hand, commercial antibiotic drugs were bought from the local drug stores with the doctor’s prescription. Specifically, four different FQ antibiotic drugs were evaluated: Cetraxal plus (3 mg·mL^–1^ CFX and 0.25 mg·mL^–1^ Fluocinolone acetonide, Laboratorios SALVAT, S.A., Barcelona, Spain), Ciprofloxacin Normon (250 mg CFX, Normon Laboratories, S.A., Madrid, Spain), Levofloxacin Aurovitas (500 mg LFX, APL Swift Services Limited, Birzebbugia, Malta), and Moxifloxacin Qualigen (400 mg MFX, Pharmathen, S.A., Attiki, Greece). For the analysis, drugs were dissolved or diluted in acetate buffer (pH 4.5) directly to make the expected concentrations fit in the experimental domain of the built model. Moreover, as there might be cases in the clinical use where multiple antibiotics are used in combination, or for quality control purpose during drug production, the above antibiotic drugs were analyzed both individually or in mixtures. On the other hand, considering the urine excretion rate of FQ antibiotics,^25^ the analysis of FQs in human urine was also attempted. Urine samples were collected in sterile bottles from volunteers, diluted 40 times with acetate buffer (pH 4.5), and then spiked with the FQ stocks. Again, the dilution of urine samples was required so as to ensure that the concentration falls within the experimental domain of the model. All real samples were measured under the same conditions as already described, without any further pretreatment.

### Data Processing

Multivariate data analysis was carried out in Matlab 7.1 (MathWorks, Natick, MA, USA) by means of specific routines developed by the authors, employing its Statistics and Neural Network toolboxes. The voltammetric responses of the sensor array were combined and compressed by employing discrete cosine transform (DCT), which allowed to reduce the dimensionality of the input signals while preserving the relevant information.^26^ Next, the obtained coefficients were either submitted to principal component analysis (PCA) or artificial neural networks (ANNs).

## Results and Discussion

### Design of an MIP(FQs)@Au-fMWCNT-Based ET

The main goal of this study is the development of a sensitive ET for the simultaneous identification and quantification of FQs (Figure S2). In this regard, the fabrication of MIP-based electrochemical sensors toward FQs (MIP(FQs)) is based on the electropolymerization approach. To further improve the performance of the MIP-based sensors, fMWCNTs that have high conductivity and Au NPs that have electrocatalytic properties were incorporated before the electropolymerization of MIP(FQs) films, as depicted in Scheme S1a. Upon synthesis of the different MIPs, the sensors were combined into a sensor array to achieve the simultaneous determination of the three FQs in the mixtures (Scheme S1b).

One of the key parameters for MIP-based sensors is the thickness of the MIP films, which is critical for mass and electron transport. If electropolymerization is carried out under potentiostatic conditions, the thickness of the film can be easily controlled by adjusting the deposition charge. However, in our case, polymerization was carried out by CV under nonstirred conditions, where the mass transfer is controlled by the diffusion process and the creation of the molecular imprints is favored by the diffusion of the electroactive template toward the electrode, generating a far higher number of recognition sites.^27,28^ Thus, the film thickness was optimized by adjusting the number of cycles of the electropolymerization in the range from 5 to 35 cycles. As shown in Figure S3a, the current peak of MIP(CFX)@Au-fMWCNTs is higher when 10 cycles were selected, as thicker films cannot ensure an efficient electron and mass transport. Moreover, ensuring a sufficient number of specific sites is critical for in the specific recognition of the template by the MIP. While a low concentration of the template might lead to poor imprinting, a too large concentration is not good for the formation of the polymers. To this end, the molar ratio of Py to CFX in the electropolymerization solution was also optimized (Figure S3b), from which a ratio of 5:1 was selected as optimal.

### Morphological Characterization of MIP(FQs)@Au-fMWCNT Sensors

**Figure 1:**
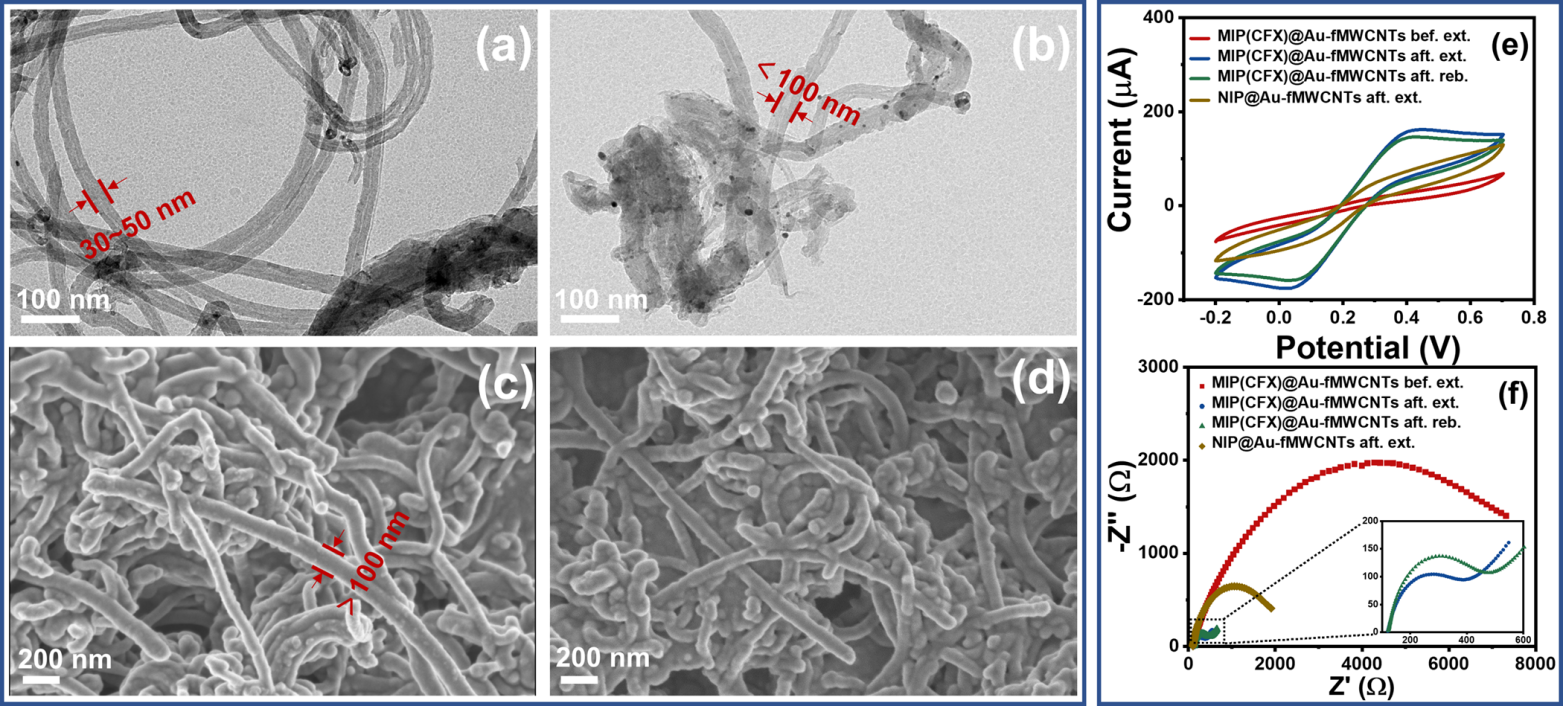
TEM images of (a) fMWCNTs and (b) Au-fMWCNTs. SEM images of MIP(CFX)@Au-fMWCNTs on GEC (c) before and (d) after the extraction of the template. Electrochemical characterization of the different steps involved from the preparation of the sensors to the actual sensing by means of (e) CV and (f) EIS employing a 5 mM [Fe(CN)_6_]^3–/4–^ solution in PBS: (from top to bottom) before and after extraction of the template; MIP rebinding; and NIP after the extraction process.

Both SEM and TEM were used to characterize the morphology of the fabricated nanomaterials. Figure S4a shows the SEM top view image of the bare GEC electrode, showing a smooth and uniform surface, which is in contrast to the images after Au-fMWCNT casting (Figure S4b) and electropolymerization (Figure S4c), undoubtedly confirming the modification of the electrode. As mentioned earlier, the combination of MWCNTs and Au NPs with MIP films allows obtaining a synergistic effect that results in an improvement of the sensor’s performance. Figure 1a,b shows the TEM images of fMWCNTs before and after their decoration with Au NPs, from which it can be seen that the diameter of the CNTs in both cases is certainly smaller than 100 nm. Furthermore, the surface of the CNTs seen in Figure 1a is smooth and without particles on them, while the CNTs in Figure 1b are clearly decorated with small particles on their surface, corresponding to the Au NPs.

Since different MIPs were obtained by introducing different FQ molecules during the electropolymerization process, the MIP(CFX) film grown onto Au-fMWCNTs will be presented as an example. The morphology of the MIP(CFX)@Au-fMWCNTs before and after the extraction of the template molecule was characterized by SEM, as shown in Figure 1c,d, respectively. From Figure 1c, it can be seen how the diameter of Au-fMWCNTs increased significantly due to the growth of the MIP film on the surface as this is now larger than 100 nm. It is worth noting that a thin and uniform MIP film grew along the surface of the Au-fMWCNTs instead of integrally aggregating as a top layer that covers the whole. The peculiar morphology is attributed to the electropolymerization method employed, which not only allows a better control over the distribution and thickness of the MIP film but also facilitates surface imprinting and formation of a core–shell structure.^29^ Finally, from the comparison of Figure 1c,d, it can be seen how there are no significant morphological changes in the MIP(CFX)@Au-fMWCNT structure after the extraction of the template and immersion in the EtOH–NaOH mixture, confirming the stability of the developed nanocomposite material. Moreover, the SEM image of the NIP@Au-fMWCNTs (Figure S4c) also shows a similar morphology, confirming the expected similarities between the different synthesized MIP and NIP films.

### Electrochemical Characterization of MIP(FQs)@Au-fMWCNT Sensors

**Figure 2:**
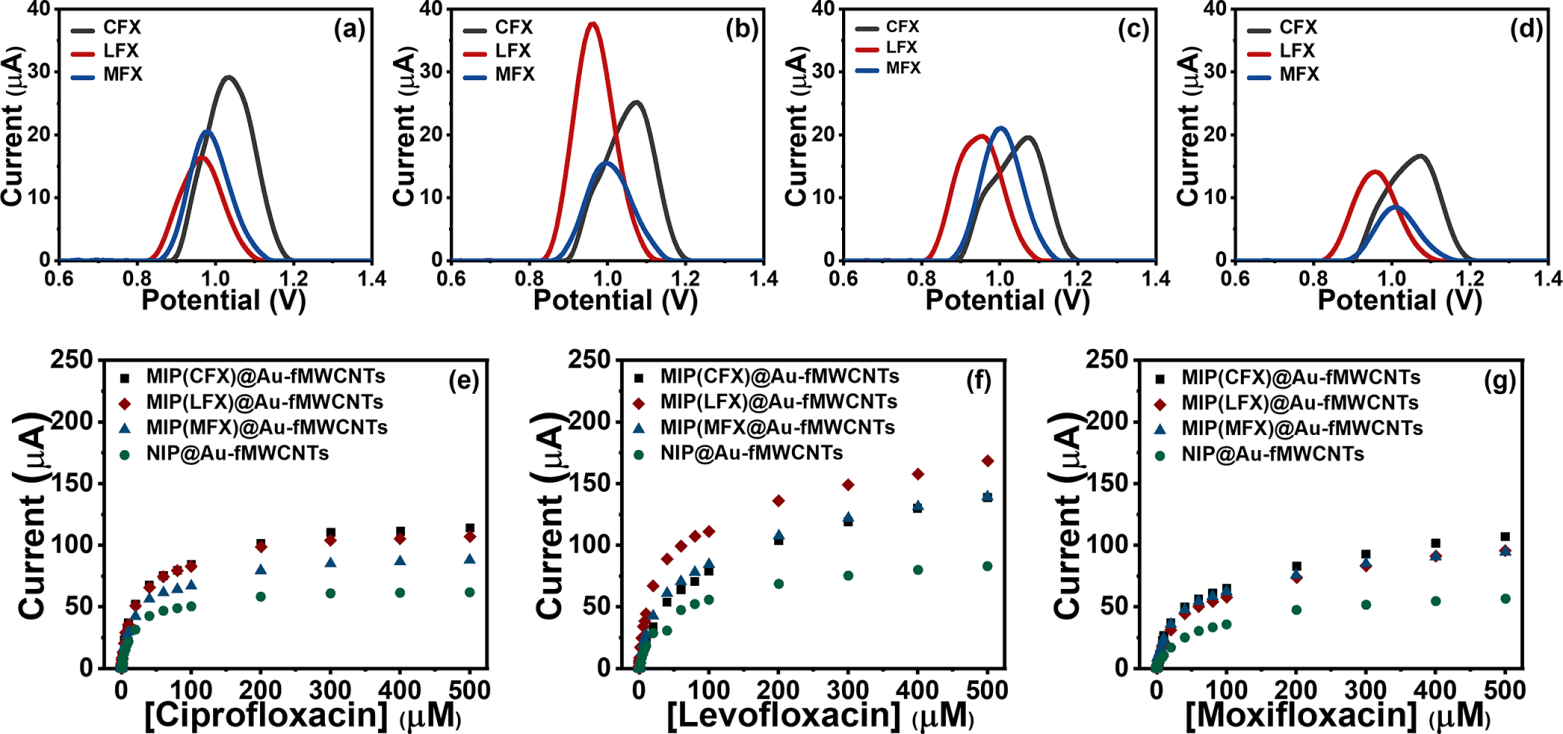
(a–d) Baseline-corrected DPV curves of (a) MIP(CFX)@Au-fMWCNTs, (b) MIP(LFX)@Au-fMWCNTs, (c) MIP(MFX)@Au-fMWCNTs, and (d) NIP@Au-fMWCNTs toward 10 μM solutions of: (black) CFX, (red) LFX, and (blue) MFX. (e–g) Calibration plots of the three target FQs (e) CFX, (f) CFX, and (g) MFX with the MIP- and NIP-based sensors.

The electrochemical behavior of the sensors was investigated by CV and EIS in a 5 mM [Fe(CN)_6_]^3–/4–^ solution. First, the response of the bare GEC was evaluated (Figure S5a), from which the reversible redox peaks of [Fe(CN)_6_]^3–/4–^ were observed. Next, this was compared to the responses of fMWCNT- and Au-fMWCNT-modified GECs, demonstrating that loading of fMWCNTs with Au NPs enhanced the electron transfer, which is reflected by the larger peaks in CV and a much lower charge-transfer resistance in EIS. Subsequently, an analogous comparison was conducted between the bare MIP sensor and that incorporating Au-fMWCNTs (Figure S5c,d). As it can be seen, MIP(CFX)@Au-fMWCNTs provided a better electrochemical performance than MIP(CFX), but worse than Au-fMWCNTs (Figure S5c,d). This is due to the poor electrical conductivity and blocked electron transport originated in the insulating features of the polymeric matrix (Figures 2 and S5). After the extraction of the template, we can observe a significant improvement in the electrochemical behavior for the MIP sensor, both in the CV and EIS analyses, because the sites created during the extraction process facilitate electron and mass transport, while the NIP sensor still showed quite a resistive behavior. Lastly, rebinding of these sites in the MIP film with the template was evaluated, and the sluggish diffusion of [Fe(CN)_6_]^3–/4–^ due to the “gate-controlled effect” is clearly reflected in the graph. Consequently, taking the above results from CV and EIS, the suitability of the developed MIP-based electrochemical sensor was confirmed.

### Optimization of the Measuring Conditions

In order to enhance the voltammetric responses of the sensors, the measuring conditions of FQs were optimized. As previously stated, the response of the MIP(CFX)@Au-fMWCNT sensor is shown as an example, taking the peak height from the DPV measurement toward a stock solution of CFX as the parameter to maximize. First, the influence of the pH of the buffer was investigated in the range from 3.5 to 5.5 (Figure S6), obtaining pH 4.5 as the most suitable.

Second, the incubation time was also optimized as an essential factor for the binding of the target analytes into the MIP film sites. Figure S7a shows how the response of the MIP-based sensor increases with the incubation time, until reaching a maximum at 300 s, indicating that the saturation had been reached. In addition, the time of the electrochemical treatment required for the sensor’s regeneration after the sensing was also studied, observing that for the measurement of a 10 μM CFX solution, 30 s were sufficient to recover the baseline (Figure S7b). However, to ensure that proper regeneration was achieved even when higher target concentrations were tested, 90 s were chosen as the regeneration time for further experiments.

Lastly, the stability of the sensors under those conditions was evaluated to ensure that they could withstand a significant number of measurements necessary for any analytical application, but especially when dealing with ETs. To this aim, CFX was selected as the substance to evaluate the variation on its voltammetric response upon successive measurements, assuming that a similar behavior will be obtained for the rest of the drugs. Employing three different electrodes, a 5 μM stock solution was measured for 25 consecutive times while measuring also a blank (acetate buffer solution) in between each measurement to evaluate the repeatability of the sensors. Thus, each sensor was used for 50 consecutive measurements. Under these conditions, the obtained relative standard deviation (% RSD) of the measured peak height corresponding to CFX was 8.7% between the different sensors over all the measurements.

### Calibration of MIP(FQs)@Au-fMWCNTs to FQs

One of the fundamental steps when developing sensors is the characterization of their response in terms of linearity, sensitivity, limit of detection (LOD), reproducibility, etc. Furthermore, when developing an ET, it is also important to assess the cross-responses toward the different target analytes. To this aim, solutions with increasing concentrations of different FQs were analyzed using the different sensors by DPV under the optimized conditions. An extract of the voltammetric responses is provided in Figure 2a–d (extracted from Figure S8a–d), where it can be seen how the three different MIP-based sensors show a voltammetric response to the three FQs (CFX, LFX, and MFX, respectively) and so does the NIP-based sensor. However, the differences in the peak position and height should be noted. As anticipated, the NIP@Au-fMWCNT-based sensor shows the lowest response to the three FQs compared with the other three MIP-based sensors due to the lack of recognition sites toward the different FQs. Furthermore, when comparing the responses of the specific MIP(FQ)@Au-fMWCNT-based sensors to the different FQs, it can be found that each MIP shows the highest response to its specific template (Figure 2). That is, MIP(CFX)@Au-fMWCNTs clearly shows the highest peak to CFX, while MIP(LFX)@Au-fMWCNTs shows to LFX, confirming the specific recognition of the different MIP(FQ) films. The fact that the MIP(MFX)@Au-fMWCNT response to MFX is not comparatively larger is attributed to the intrinsically higher electrochemical response of the other FQs, as is seen for the NIP sensor. In this regard, it can be deduced that the similar structure and nature of FQs, all based on different substitutions made to the quinolone ring, hinder the attainment of a highly specific MIP-based sensor, but lead to a class-selective material rather than a highly specific one—a situation that is well suited for ET approaches, as chemometrics may improve its specificity. Indeed, this cross-sensitivity observed is completely analogous to that of the “dummy” MIPs, where a relatively similar molecule to the target analyte is used for imprinting, although later the MIP is used not to analyze the template molecule but the target analyte.^30^

To further confirm the behavior of the MIP, the response of MIP(CFX)@Au-fMWCNTs was compared to those of the bare electrode and an electrode modified with Au-fMWCNTs, but not MIP (Figure S9). Furthermore, the response toward an antibiotic from a different family (vancomycin) as well as other types of compounds that could also be found either in tablets or urine such as glucose, ascorbic acid, and paracetamol were also evaluated. From those findings, it can be seen how the MIP contributes to increase the voltammetric responses obtained for FQs, while the response for other potentially interfering compounds is decreased. Additionally, it has to been remarked, that apart, these current maxima may appear at different potential, therefore facilitating its discrimination.

After preliminary assessment of the responses of the different sensors toward the different FQs, appropriate calibration of each MIP(FQs)@Au-fMWCNT and NIP@Au-fMWCNT was carried out. From Figure S10, it can be seen how the peak currents increase steadily when FQs concentrations become greater. The observed peaks are attributed to the oxidation of the piperazine ring common to the different FQs (Figure S11).^31,32^ A hyperbolic response was observed as evidenced in Figure 2e–g, from which the dissociation constant (*k*_D_) and the maximum binding response at saturation (*B*_max_) were calculated by fitting those to a one site saturation ligand binding model (Langmuir model, Table S1). Furthermore, a logarithmic relationship could be employed between the concentrations and the corresponding peak heights for low concentrations (from which the LOD could be calculated). In all the cases, a good linear relationship for the three FQs is obtained with *R*^2^ values close to 1.

### Qualitative Analysis of FQs

Despite MIP-based sensors are meant to be highly specific, those might still show certain cross-response when analogous molecules are analyzed, for instance, when analyzing different FQs. One of the common solutions to the limited selectivity may be to synthesize more advanced MIP-based materials. For example, in this case, limited selectivity for FQs may be partially attributed to the use of electropolymerized Py instead of a more sophisticated polymerization method employing carefully screened monomers and cross-linkers. However, the truth is that a similar behavior has also been reported with other polymerization strategies.^33,34^ In this direction, herein we propose the combination of chemometrics with MIPs as a straightforward solution to further improve its selectivity. Such an approach allows to shift the complexity from the chemical to the data processing side; while in turn further advantages might also be attained, that is, to cope with nonlinear systems or to counterbalance possible matrix effects.^9,35^

**Figure 3:**
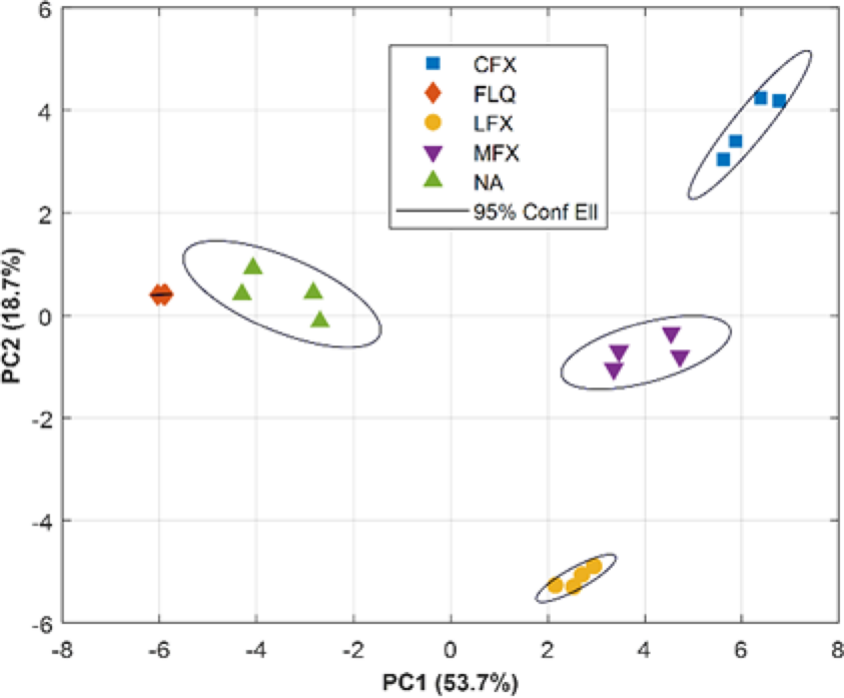
Score plot obtained from DCT–PCA of the voltammetric responses of the MIP-based ET toward five different FQs. Ellipses plotted correspond to 95% confidence limits for each of the clusters.

In order to demonstrate such a statement, the analysis of stock solutions of five different FQs was carried out under the optimized conditions, employing the three developed MIP- and NIP-based sensors. Next, the voltammetric responses were compressed by means of DCT from 114 points down to 12 coefficients with high fidelity as evidenced from Figure S12 and submitted to PCA (Figure 3). First, it should be noted that *ca.* 72.4% of the original data variance is now represented by only those two first principal components (PCs), providing a better representation of sample measurements that allows an easier assessment of its (dis)similarities. Second, we can see how different clusters corresponding to each of the FQs appear and how these clusters separate from each other. Consequently, the superb ability of the developed ET system to discriminate FQs is well denoted.

### Quantification of FQs

In view of the above results, the quantification of mixtures of different FQs was attempted, namely, mixtures of CFX, LFX, and MFX. To this aim, the set of samples described in the Experimental Section was analyzed, and, as before, the obtained responses were compressed by means of DCT. In this case, ANNs were chosen for data modeling, making use of the samples of the training subset to optimize the topology of the neural network, while those of the test subset were used to assess the model performance. In this manner, we ensured a more realistic assessment as different data subsets are used for each step.

**Figure 4:**
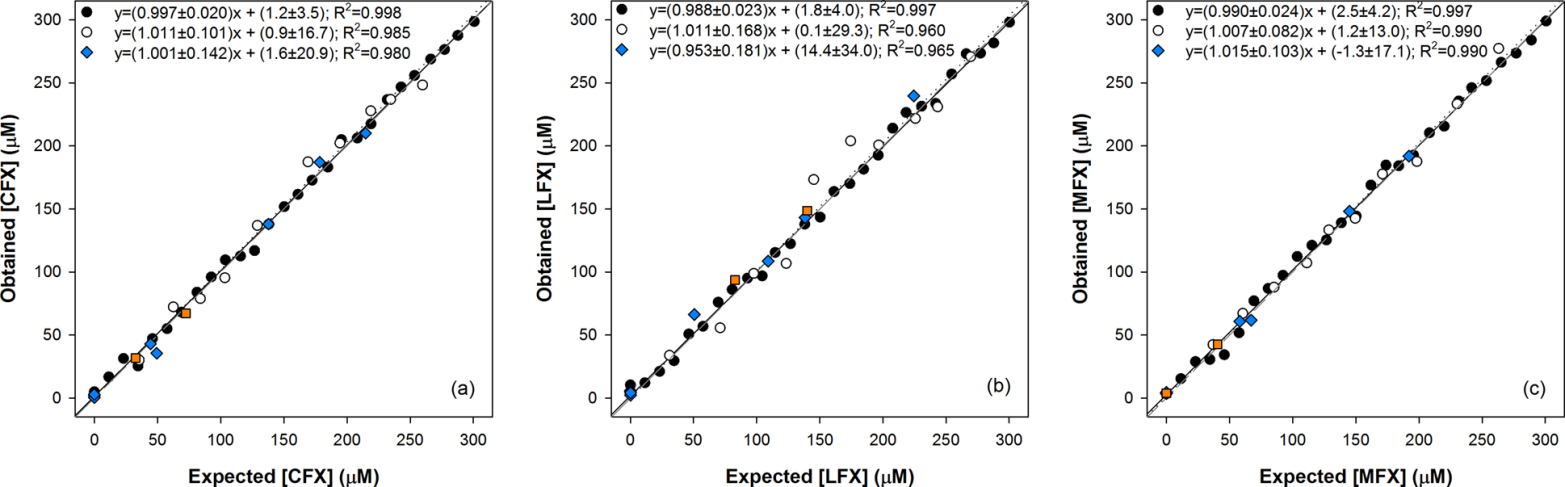
Modeling ability of DCT–ANN. Comparison graphs of obtained versus expected concentrations for (a) CFX, (b) LFX, and (c) MFX, for both the training (solid circle, solid line) and testing (open circle, dotted line) subsets. The dashed line corresponds to the ideal comparison line (*y* = *x*). The results of the analysis of the pharmaceutical samples (solid diamond, blue) and spiked human urine samples (solid square, orange) are also plotted.

After an iterative trial-and-error process in which different neural network parameters were tuned, the final architecture had 48 input neurons (12 coeffs. × 4 sensors) in the input layer, 7 neurons and *logsig* transfer function in the hidden layer, and 3 neurons and *tansig* transfer function in the output layer (one for each of the FQs under study). Subsequently, comparison graphs of predicted versus expected concentrations were built both for the training and testing subsets to easily assess their performance (Figure 4). Moreover, the regression parameters were also calculated to numerically assess the goodness of the modeling. As can be observed from Figure 4, for each FQ, a good trend was obtained with regression lines close to the ideal ones (*y* = *x*, slope, intercept, and correlation coefficient values of 1, 0, and 1, respectively). Consequently, we confirmed the potential of the approach not only to discriminate between different FQs (as shown previously with the PCA), but also to individually quantify them even in mixtures.

To somehow assess the improvement derived from the usage of the MIP-based sensor array, the same set of samples was also measured by employing a bare GEC electrode. The recorded data were then processed in the same way, and the normalized root mean square error (NRMSE) for both approaches was compared. After optimization of the DCT–ANN topology, a total NRMSE of 0.134 was obtained for the bare electrode; a value that is significantly larger than that of the MIP-based ET, which was 0.033.

Finally, to further demonstrate the applicability of the proposed MIP-based ET, some pharmaceutical and human urine samples were analyzed. For both of them, no other sample pretreatment than its proper dilution to fit within the evaluated range of concentrations was carried out. As before, the corresponding readings of the sensors were compressed with DCT and interpolated into the built ANN model. Those are also plotted in Figure 4, where a good agreement between the expected and predicted values is also observed despite the relatively complex matrixes of the pharmaceutical tablets (excipients, fillers, disintegrants, binders, etc.) and urine (urea, inorganic salts, creatinine, uric acid, proteins, vitamins, etc.). Again, confirming the suitability and advantages derived from the combination of MIPs and ETs, which allowed to address not only the lack of selectivity of MIPs, but the successful quantification of FQs mixtures in real samples.

## Conclusions

Herein, a sensitive ET based on different MIPs incorporating Au NP-decorated MWCNTs has been developed for the analysis of FQs. In contrast to the conventional methods for MIP synthesis, in this study, MIP films were electropolymerized directly on the surface of Au-fMWCNTs. The use of electropolymerization facilitates the integration of MIPs with voltammetric sensors, while the combination of CNT’s large surface/mass ratio and high conductivity and the catalytic effect of Au NPs with the specific recognition of MIPs leads to a synergistic effect, which leads to an improved performance of the developed sensors.

The developed sensors were morphologically and electrochemically characterized to confirm the modification of the electrodes and assess their analytical responses. Despite the good performance obtained, the similar structure of the different FQs hinders the attainment of highly specific MIPs, leading to a class-selective material rather than a highly specific one. In this direction, to improve their specificity, the developed MIP-based sensors were combined into a sensor array to develop an MIP-based ET. Thanks to the use of chemometrics, it was possible not only to discriminate between different FQs, but also to achieve the simultaneous quantification of CFX, LFX, and MFX. Finally, the developed ET was applied to the analysis of pharmaceutical and biological samples, with satisfactory recovery values obtained, confirming the lack of matrix effects.

Overall, the proposed approach is an appealing and promising tool for the determination of FQs, but more importantly, it paves the way for the development of similar applications. On the one side, electropolymerization is a facile approach for the fabrication of MIP-based sensors, while the integration of nanomaterials leads to an improved electrochemical behavior. On the other side, applied machine learning provides a straightforward approach to tackle MIP’s cross-response.

## Supporting Information Available

Molecular structure of the analytes; SEM images of the sensors; voltammograms recorded during electropolymerization; optimization of the experimental conditions; characterization of the voltammetric responses; and detailed results of the data treatment (PDF)
