# Supplementary material for: A Sensor Array Based on Molecularly Imprinted Polymers and Machine Learning for the Analysis of Fluoroquinolone Antibiotics
Source: ACS Sens. 2022 Oct 25;7(11):3318–25. doi: 10.1021/acssensors.2c01260 (PMC9706806; doi:10.1021/acssensors.2c01260)
Supplement: Supplementary file 1 — se2c01260_si_001.pdf [file se2c01260_si_001.pdf]

A sensor array based on molecularly imprinted polymers and machine learning for the analysis of fluoroquinolone antibiotics

Mingyue Wang, Xavier Cetó and Manel del Valle*

Sensors and Biosensors Group, Department of Chemistry, Universitat Autònoma de Barcelona, Faculty of Sciences, 08193 Bellaterra, Barcelona, Spain

Supporting Information

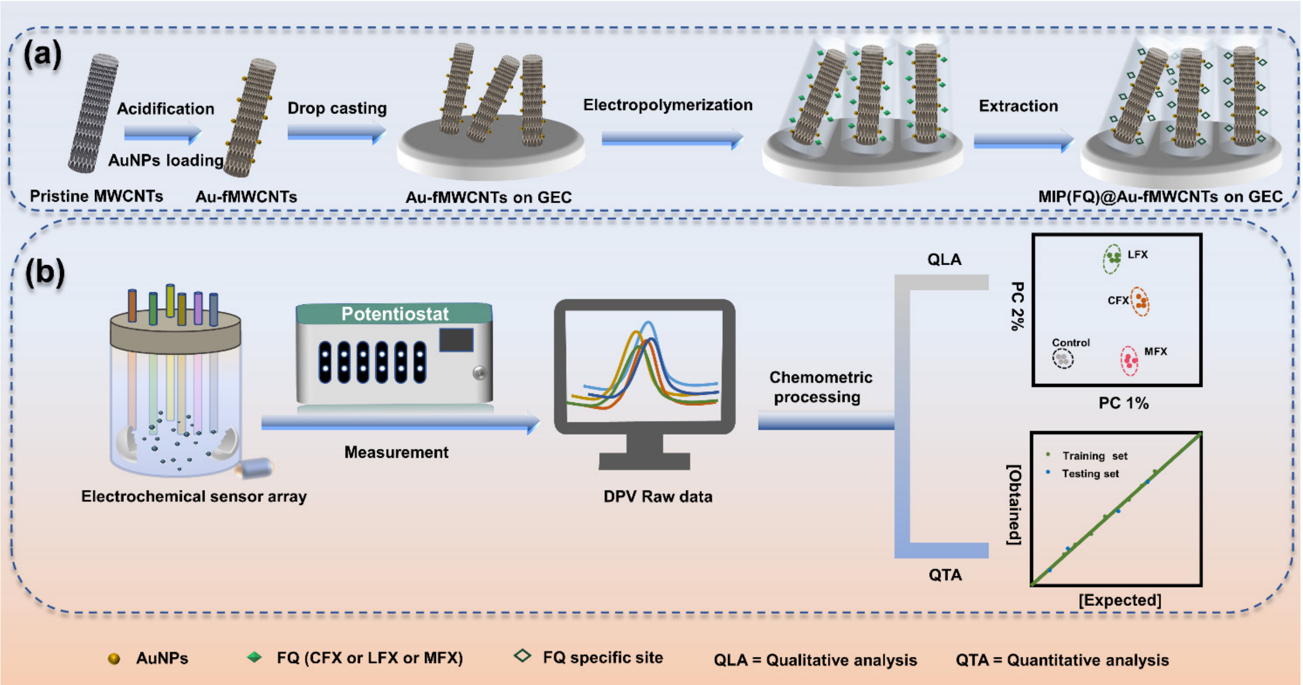

Scheme S1. Design of the ET based on MIP(FQ)@Au/f-MWCNTs: (a) Main steps related to the fabrication of the MIP(FQ)@Au-fMWCNTs sensors, and (b) combination of the sensors into an array to develop the ET for the qualitative and quantitative analysis of FQs.

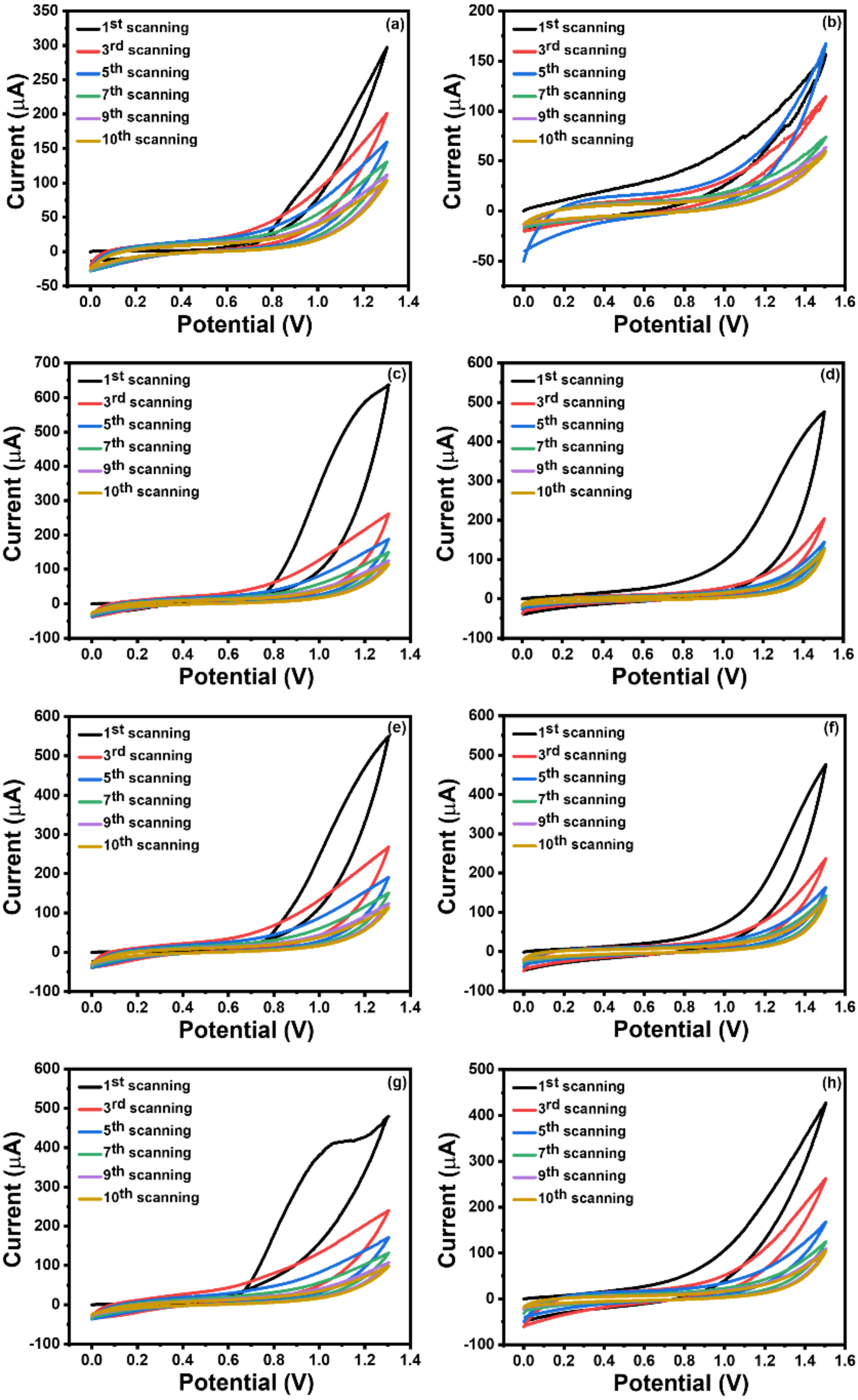

Figure S1. The CV curves on the left were recorded during the electropolymerization of MIP(FQ) films onto Au-fMWCNTs (a) with CFX, (c) with LFX, (e) with MFX and (g) without any FQ template molecules. The CV curves on the right were recorded during the extraction of FQ template molecules (b) CFX, (d) LFX, (f) MFX and (h) NIP as control for MIP(FQs)@Au-fMWCNTs.

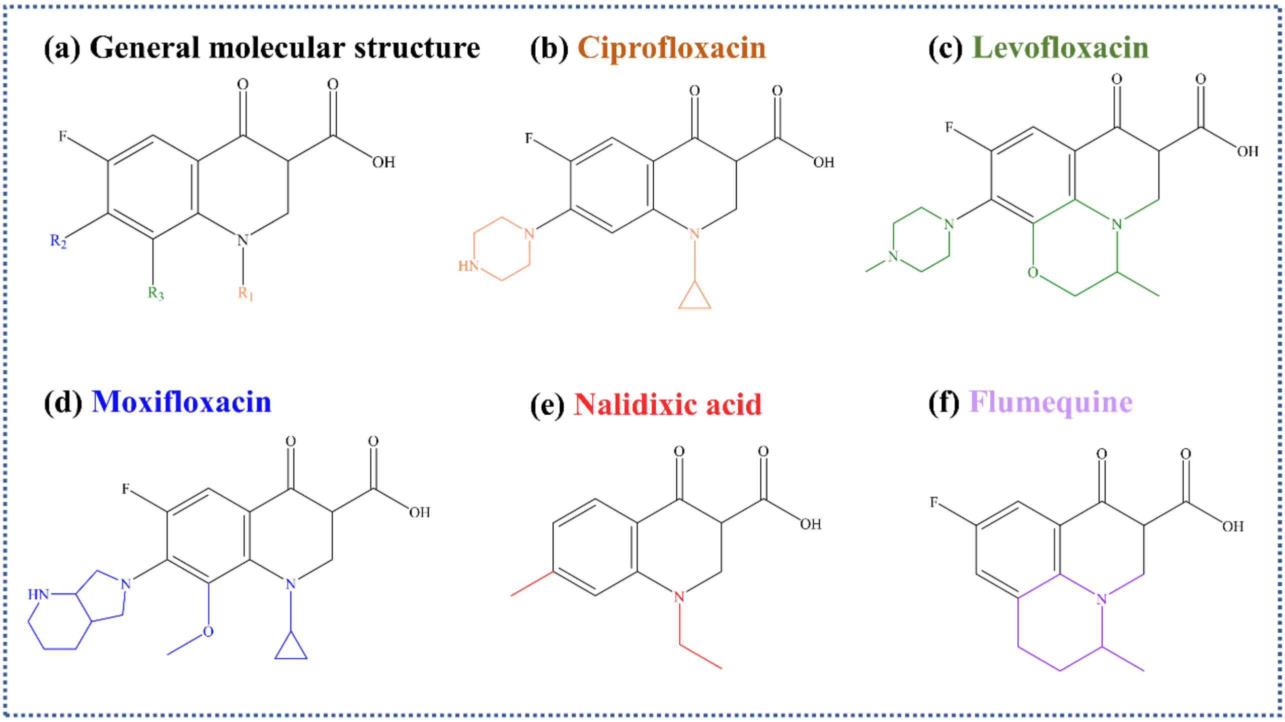

Figure S2. Molecular structure of FQ antibiotics: (a) general structure of FQs, (b) ciprofloxacin (CFX), (c) levofloxacin (LFX), (d) moxifloxacin (MFX), (e) nalidixic acid (NA) and (f) flumequine (FLQ).

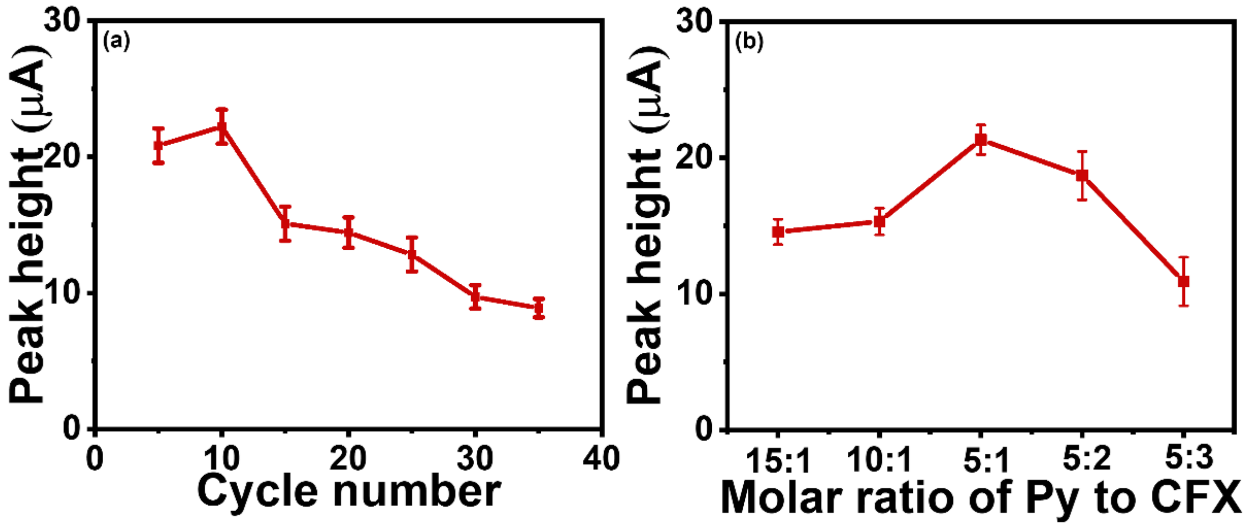

Figure S3. Effect on the sensor response of (a) the number of cycles and (b) the ratio of the monomer to template during the electropoymerization. The peak height was taken from DPV measurements with MIP(CFX)@Au-fMWCNTs sensor towards a 5 μM CFX solution. The electrolyte used to study the effect of cycle number was composed by Py and CFX at ratio of 5:1, while the scanning cycle number was fixed at 10 when investigated the effect of molar ratio of monomer to template.

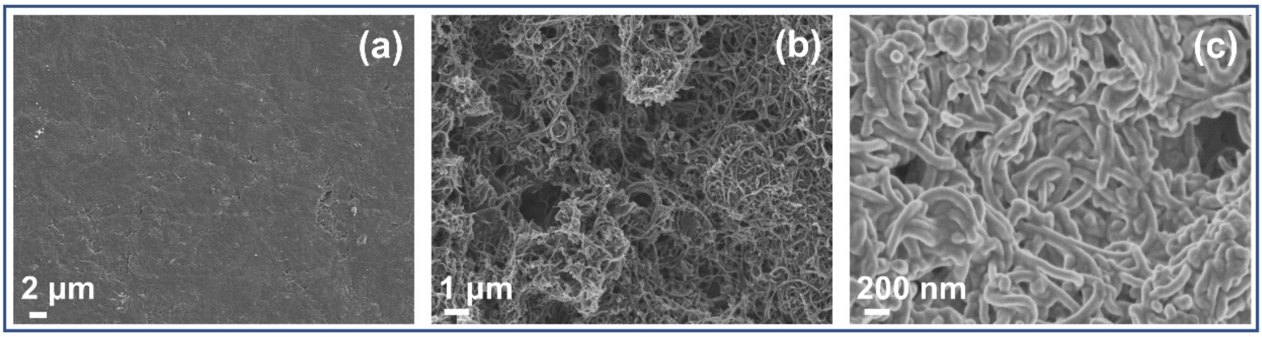

Figure S4. SEM images of (a) bare GEC, (b) Au-fMWCNTs and (c) NIP @Au-fMWCNTs.

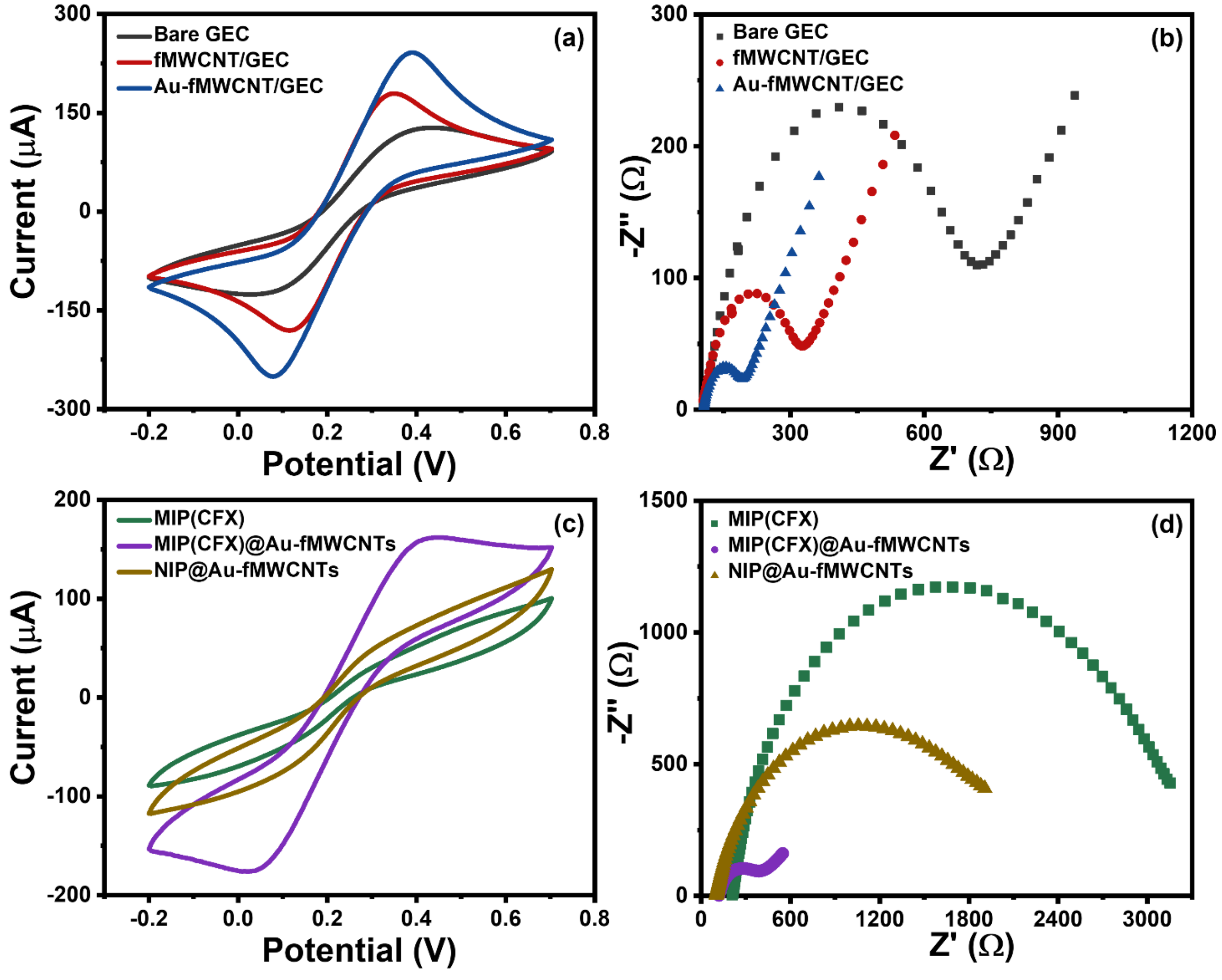

Figure S5. Electrochemical characterization of the sensor response towards a 5 mM $[Fe(CN)_6]^{3-/4-}$ solution in PBS during the different steps involved in the preparation of the sensors by (a,c) cyclic voltammetry and (b,d) electrochemical impedance spectroscopy.

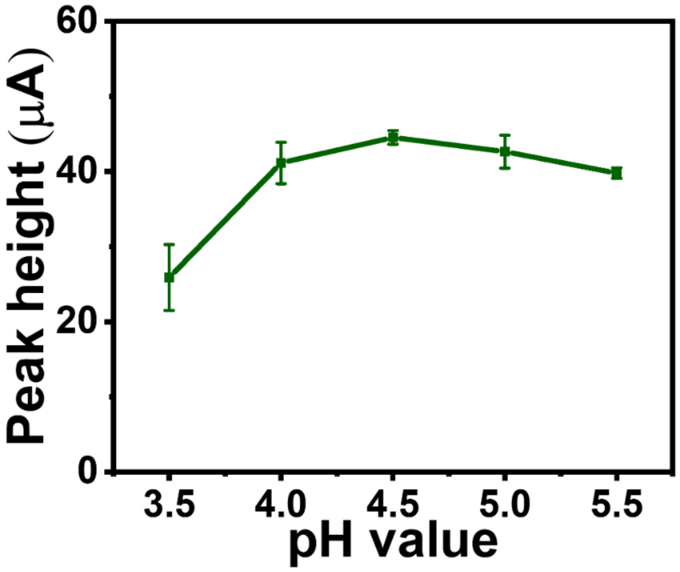

Figure S6. Effect of the pH of acetate buffer on the MIP(CFX)@Au-fMWCNTs sensor response towards a 10 μM CFX solution.

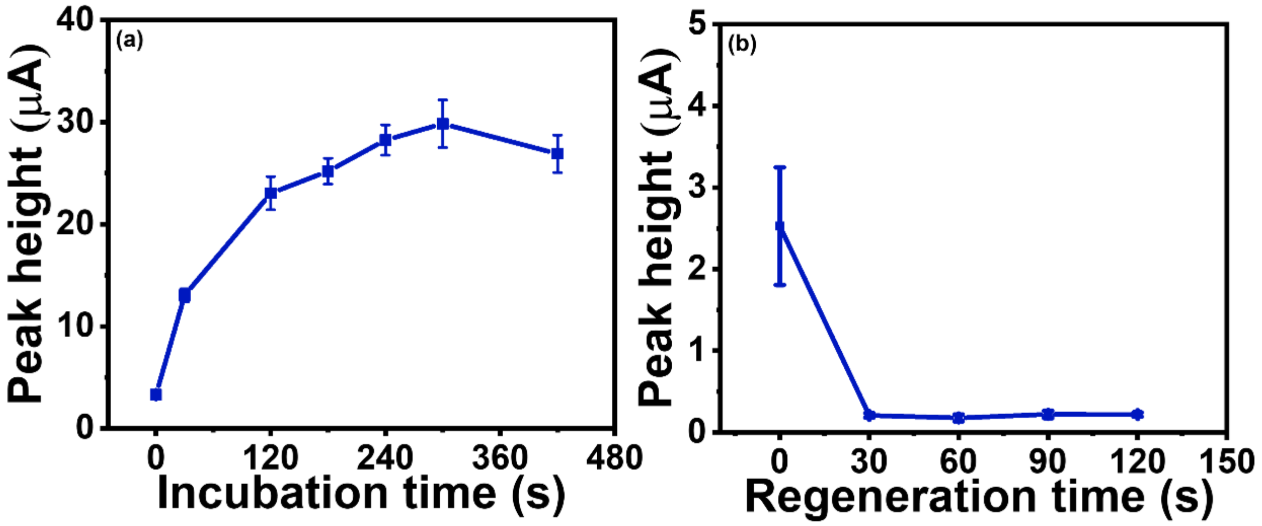

Figure S7. Effect on the sensor response of (a) the incubation time with the template and (b) the regeneration time after each measurement. The peak height was taken from DPV measurements with MIP(CFX)@Au-fMWCNTs sensor towards a 10 μM CFX solution.

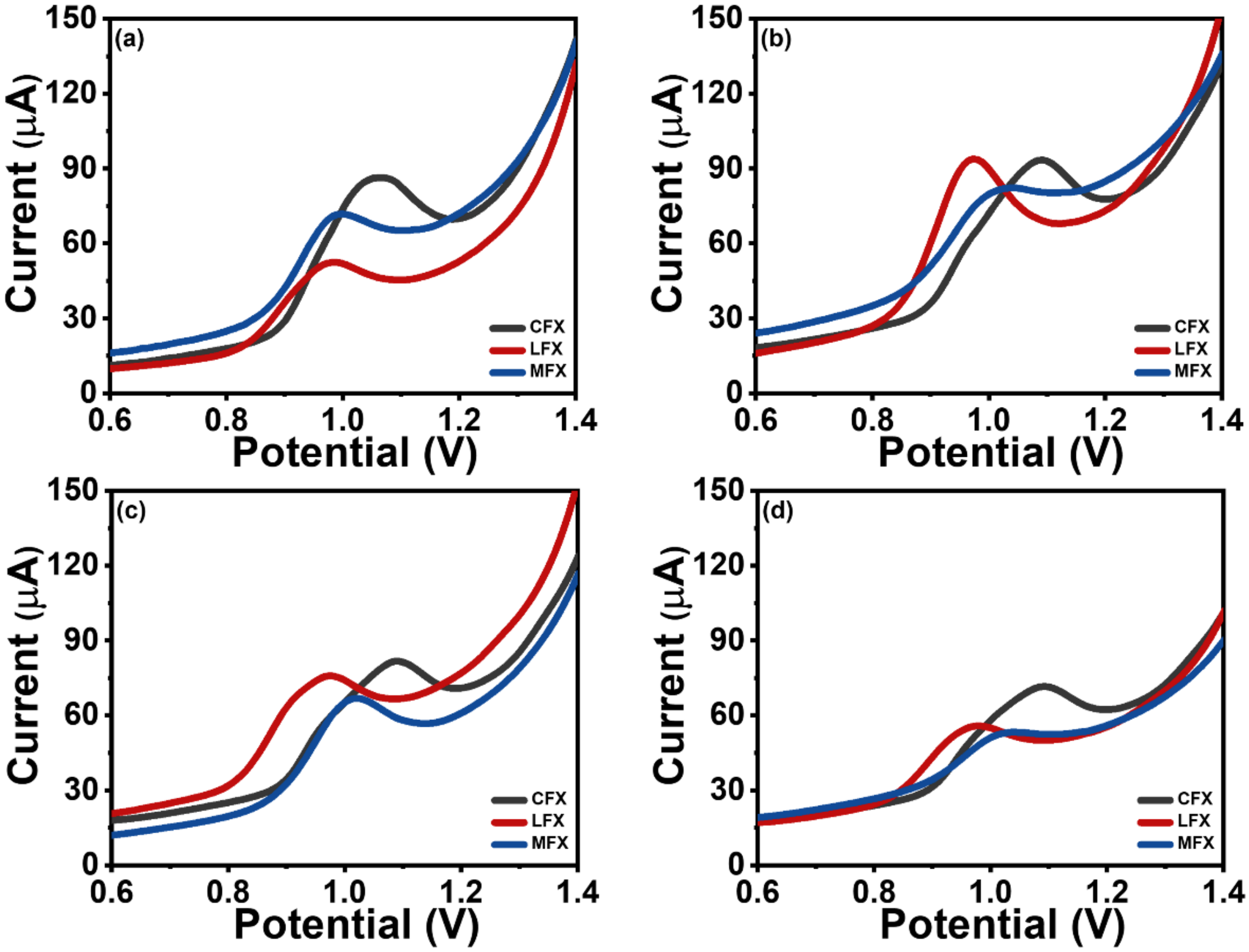

Figure S8. Raw DPV curves of (a) MIP(CFX)@Au-fMWCNTs, (b) MIP(LFX)@Au-fMWCNTs, (c) MIP(MFX)@Au-fMWCNTs and (d) NIP@Au-fMWCNTs towards a 10μM solution of: (black) CFX, (red) LFX and (blue) MFX.

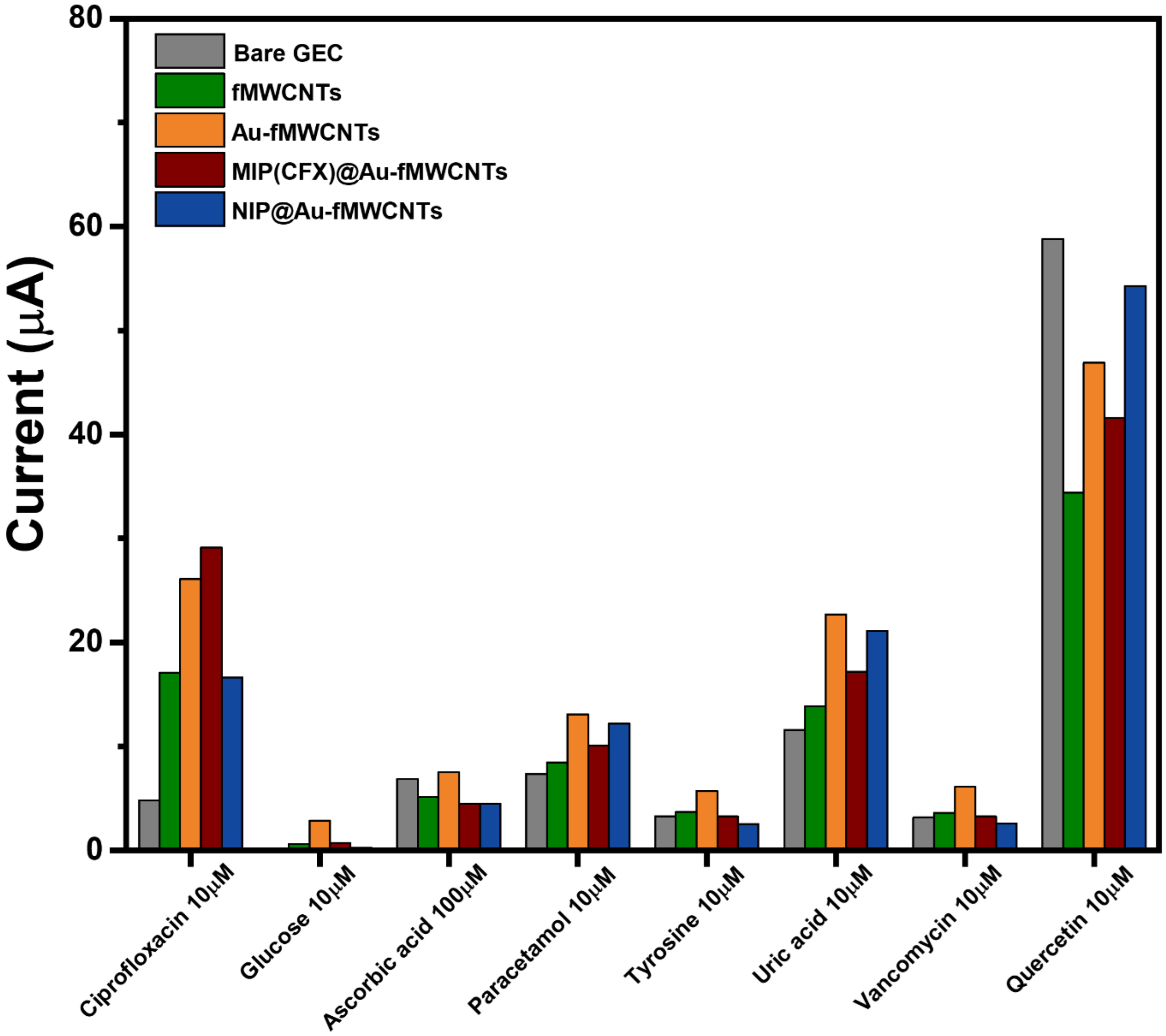

Figure S9. Comparison of the peak height calculated from the DPV measurements for different substances under the optimized conditions for each of the different modification steps.

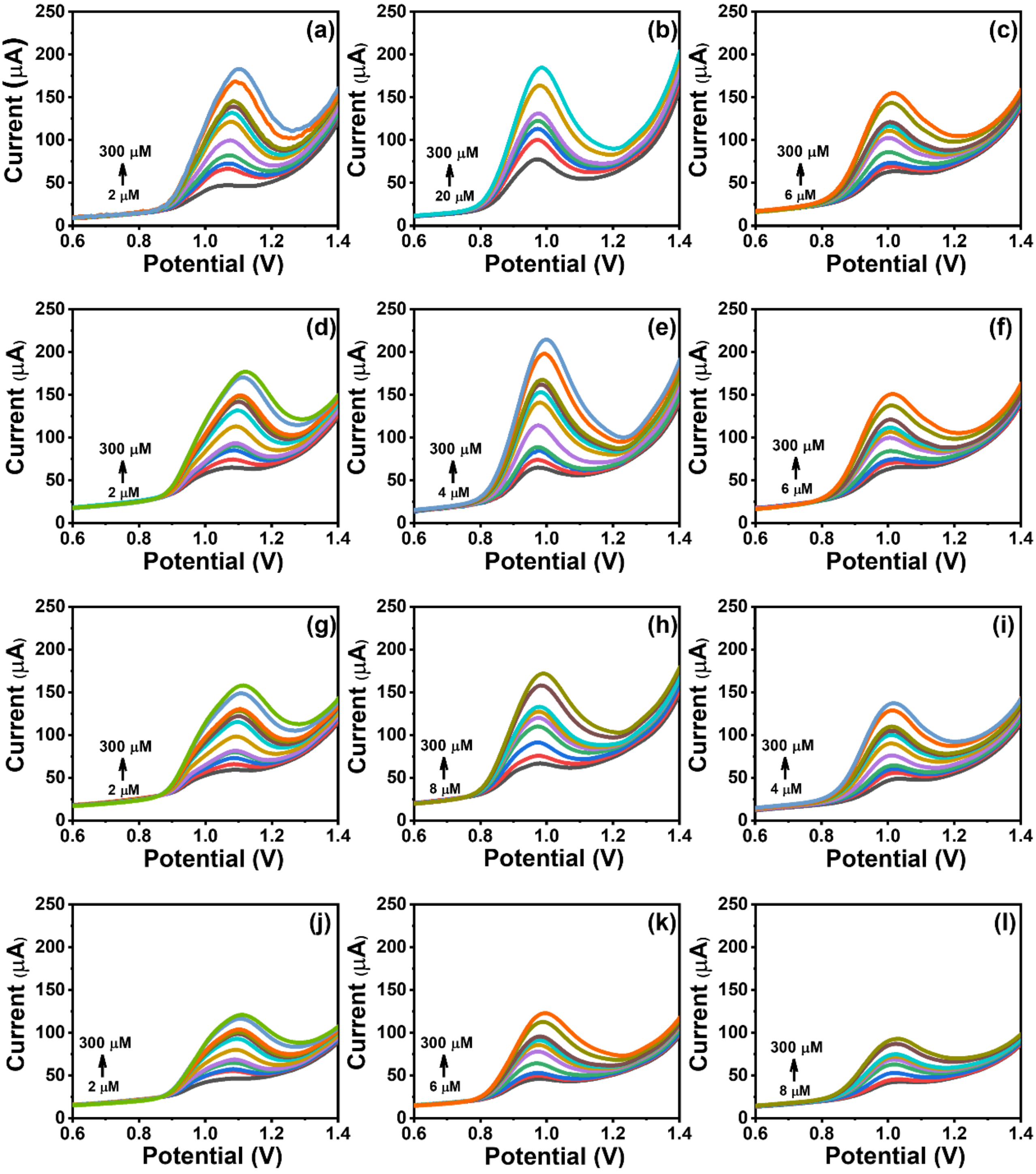

Figure S10. DPV curves in the corresponding linear concentration range of (a-c) MIP(CFX)@Au-fMWCNTs towards CFX, LFX and MFX (d-f) MIP(LFX)@Au-fMWCNTs towards CFX, LFX and MFX (g-i) MIP(MFX)@Au-fMWCNTs towards CFX, LFX and MFX and (j-l) NIP@Au-fMWCNTs towards CFX, LFX and MFX.

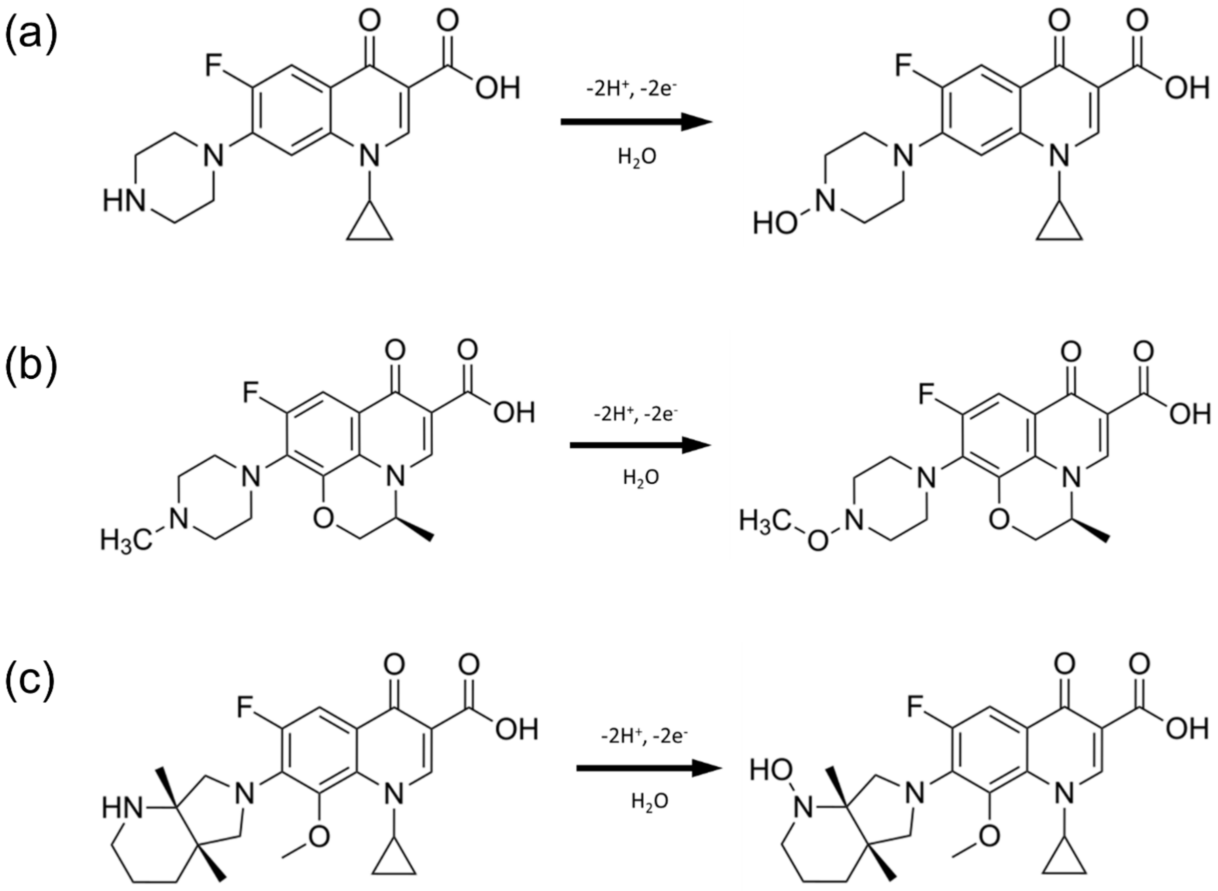

Figure S11. Electrochemical oxidation mechanism of (a) CFX proposed by de Faria *et al.*[S1], (b) LFX proposed by de Faria *et al.* [S1] and (c) MFX proposed by Akilarasan *et al.* [S2].

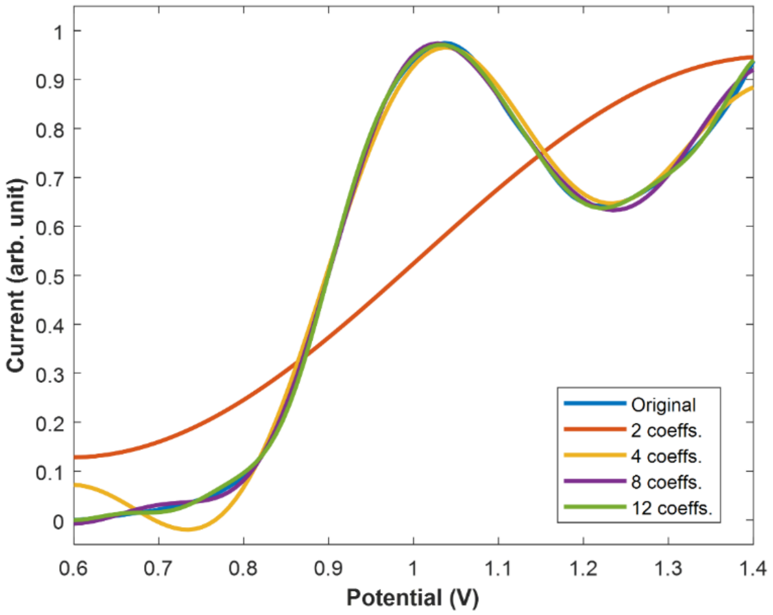

Figure S12. Effect of the number of coefficients taken during DCT compression step on the reconstruction of the original voltammetric signal.

Table S1. Analytical parameters of MIP(FQs)@Au-fMWCNTs sensors towards the three FQs calculated from DPV measurements.

Analyte	Sensor	B_{max} (μA)	K_D (μM)
CFX	MIP(CFX)@Au-fMWCNTs	114	23.4
	MIP(LFX)@Au-fMWCNTs	108	22.3
	MIP(MFX)@Au-fMWCNTs	88.1	21.7
	NIP@Au-fMWCNTs	63.2	19.5
LFX	MIP(CFX)@Au-fMWCNTs	155	3.76
	MIP(LFX)@Au-fMWCNTs	165	33.2
	MIP(MFX)@Au-fMWCNTs	148	60.9
	NIP@Au-fMWCNTs	89.0	53.6
MFX	MIP(CFX) @Au-fMWCNTs	109	49.0
	MIP(LFX)@Au-fMWCNTs	99.4	54.2
	MIP(MFX)@Au-fMWCNTs	94.5	37.3
	NIP@Au-fMWCNTs	61.8	59.9

References

[S1] de Faria, L. V.; Lisboa, T. P.; Campos, N. d. S.; Alves, G. F.; Matos, M. A. C.; Matos, R. C.; Munoz, R. A. A., Electrochemical methods for the determination of antibiotic residues in milk: A critical review. *Anal. Chim. Acta* **2021,** *1173*, 338569.

[S2] Akilarasan, M.; Tamilalagan, E.; Chen, S.-M.; Maheshwaran, S.; Fan, C.-H.; Habila, M. A.; Sillanpää, M., Rational synthesis of rare-earth lanthanum molybdate covered reduced graphene oxide nanocomposites for the voltammetric detection of Moxifloxacin hydrochloride. *Bioelectrochemistry* **2022,** *146*, 108145.
